# Supplementary material for: Decreased maternal serum acetate and impaired fetal thymic and regulatory T cell development in preeclampsia
Source: Nat Commun. 2019 Jul 10;10:3031. doi: 10.1038/s41467-019-10703-1 (PMC6620275; doi:10.1038/s41467-019-10703-1)
Supplement: Supplementary file 1 — Supplementary Information [file 41467_2019_10703_MOESM1_ESM.pdf]

Supplementary Information

Decreased maternal serum acetate and impaired fetal thymic and regulatory T cell development in preeclampsia

HU et al.

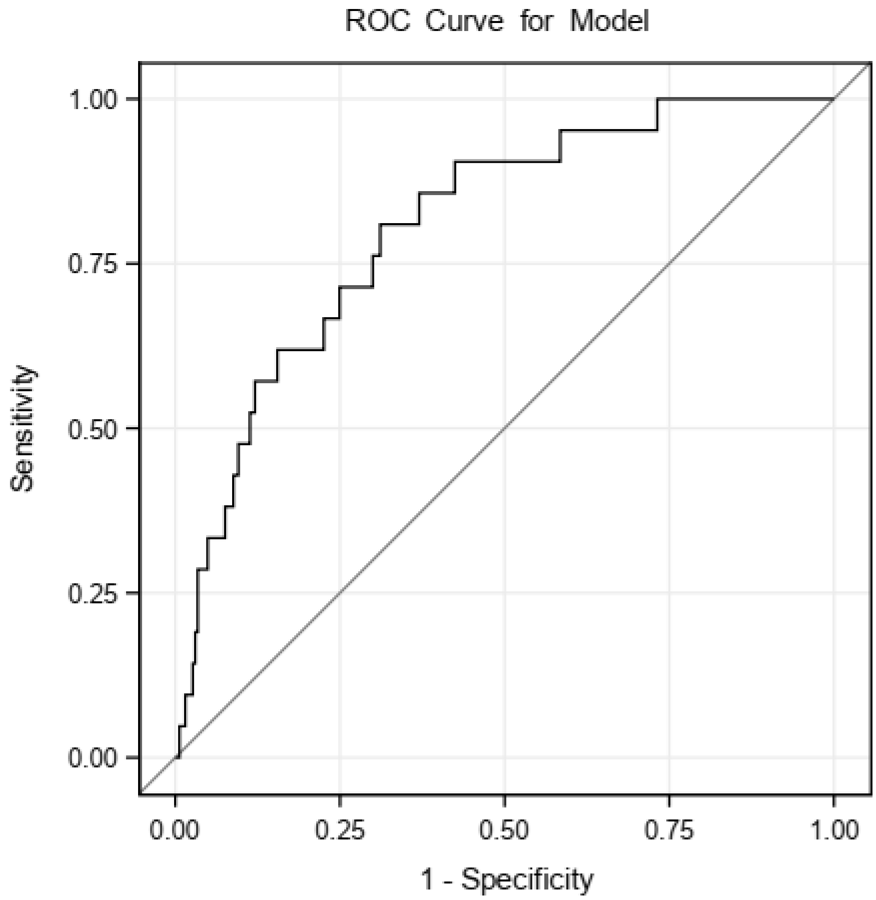

Supplementary Figure. 1 Fetal thymus diameter at mid-gestation as a predictive tool for preeclampsia.

Fetal thymus diameter at 17-22 weeks gestation was evaluated as a predictive tool for preeclampsia in a model which also included fetal head circumference at mid-gestation, gestational age and maternal BMI variables. ROC curve analysis was found to have an AUC of 0.81.

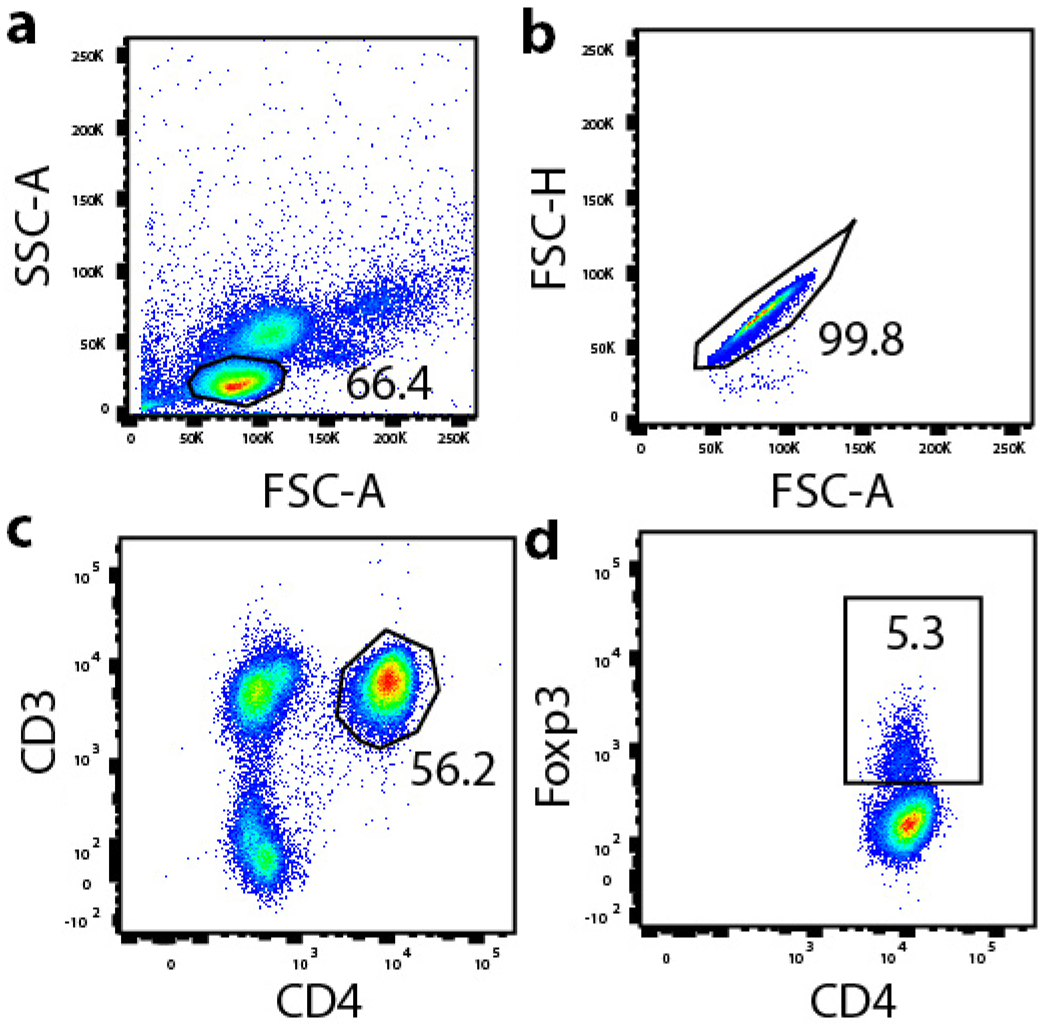

Supplementary Figure 2 (corresponding to Fig. 2a-2b). Gating strategy for analysis of Foxp3^{+} Tregs from PBMC

(a-b) Lymphocytes and single cells were gated based on forward and side scatter. **(c-d)** CD4^{+}CD3^{+} cells were identified from total lymphocytes and selected for the expression of Foxp3.

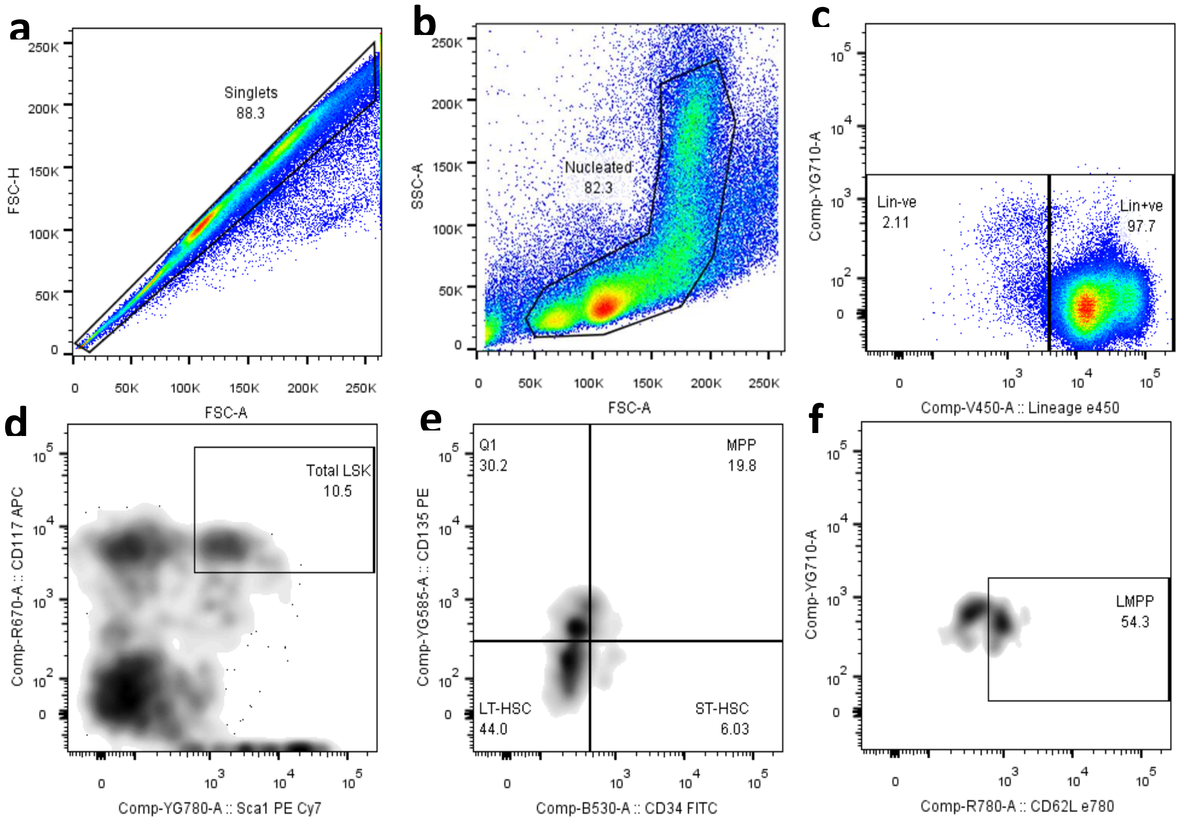

Supplementary Figure 3 (corresponding to Fig. 6d). Gating strategy to identify bone marrow lymphoid-primed multipotent progenitors (LMPP)

(a-b) Forward versus side scatter plots were used to select nucleated cells and exclude cellular debris. **(c)** Bone marrow cells were gated to exclude all lineage positive (Lin+ve) markers which includes a combination of the surface markers NK1.1, CD3e, CD4, CD8a, CD11b, CD19, Ly6G, TER119, CD11c and CD45R/B220. **(d)** Total Lin-Sca+Kit+ (LSK) cells were selected based on positive expressions of CD117 and Sca1. **(e)** The LSK population was further divided into long-term hematopoietic stem cells (LT-HSC), short-term hematopoietic stem cells (ST-HSC) and multipotent progenitors (MPP) based on differential expressions of CD135 and CD34. **(f)** LMPP was identified based on the positive expression of CD62L.

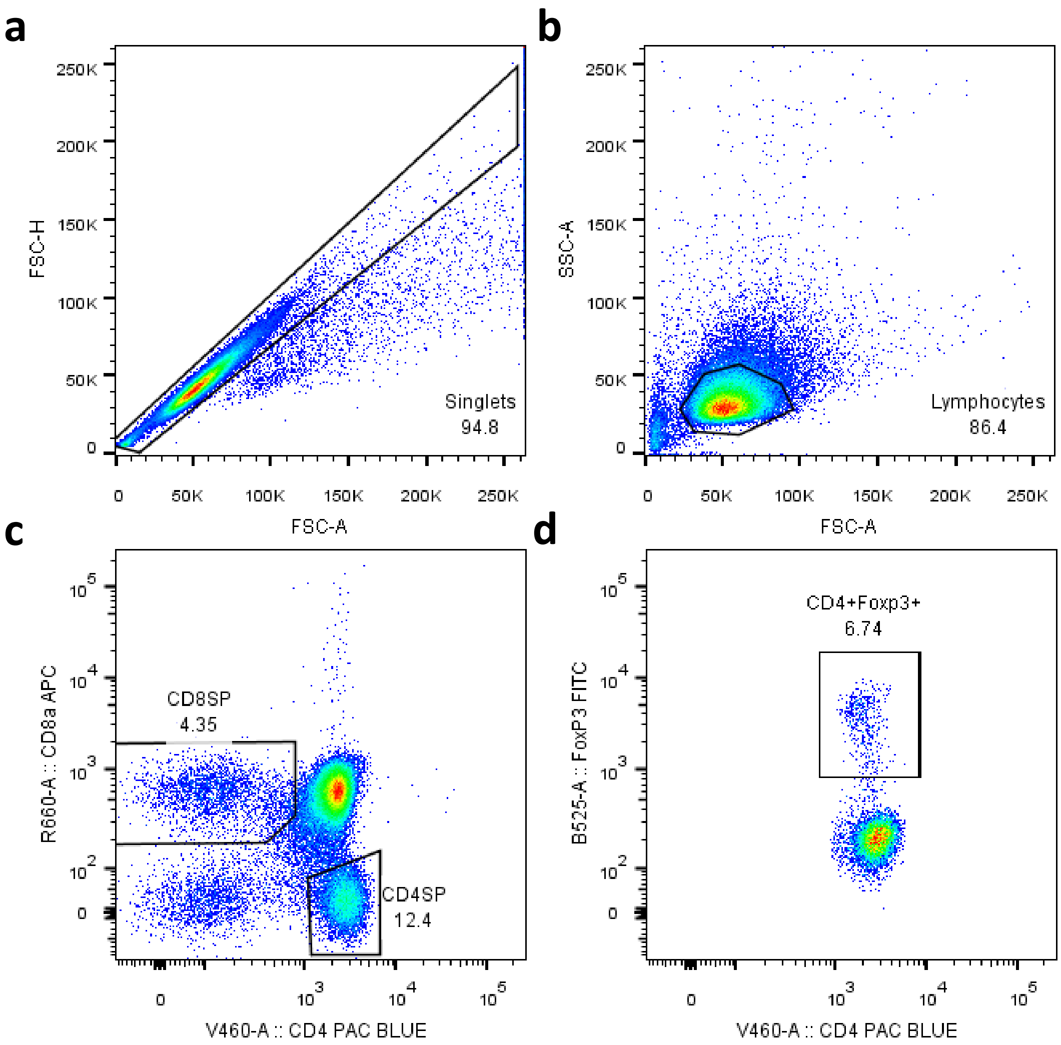

Supplementary Figure 4 (corresponding to Fig. 6e & f). Gating strategy for analysis of Foxp3⁺ Tregs from thymus

(a-b) Single cells and lymphocytes were gated based on forward and side scatter. **(c)** CD8 single positive (CD8SP) and CD4 single positive (CD4SP) cells were identified from total lymphocytes. **(d)** CD4SP cells were selected for further analysis and examined for the expression of Foxp3.

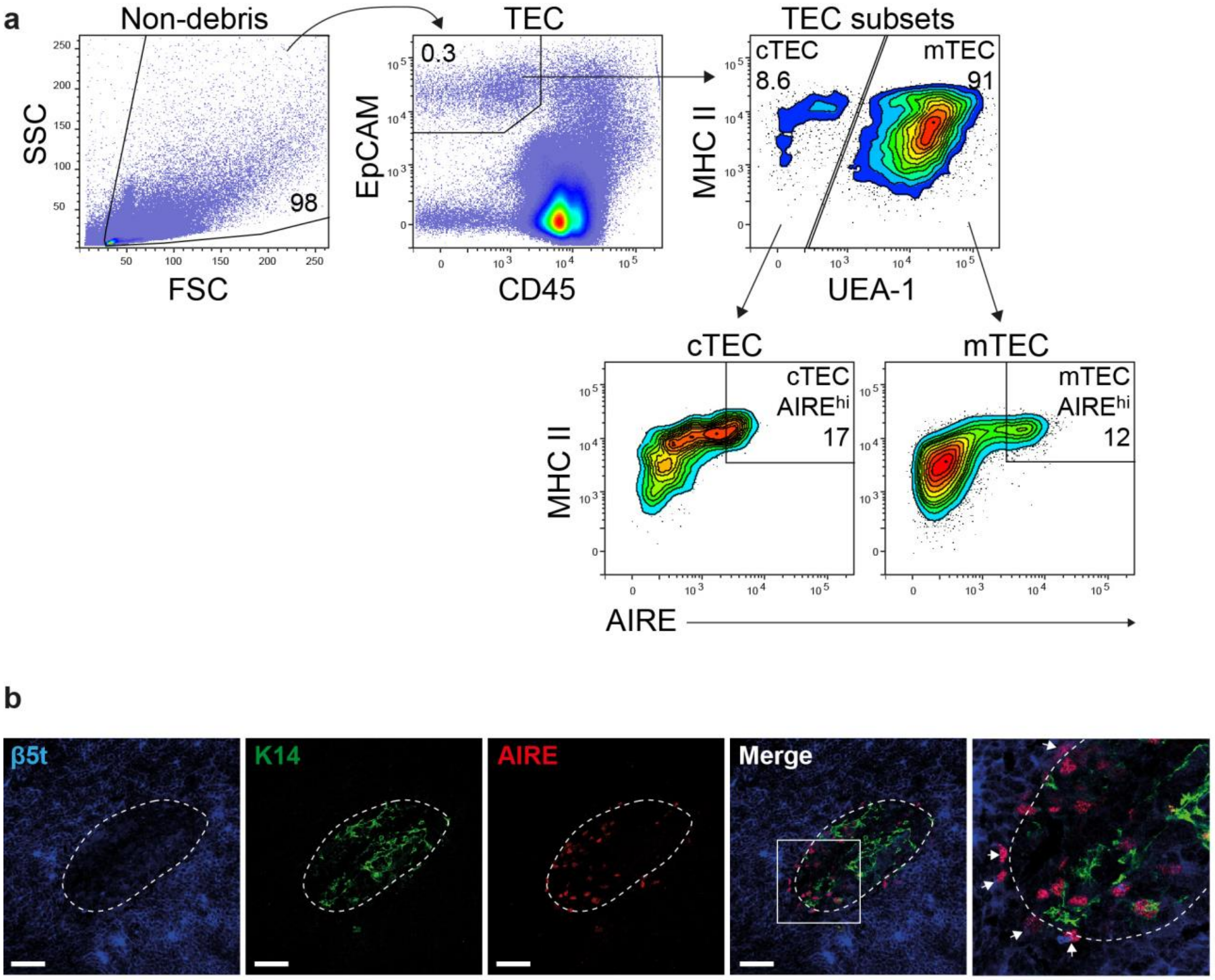

Supplementary Figure 5. Thymus Expression Levels of the Autoimmune Regulator (AIRE) Protein

(a) Gating strategy for flow cytometry analysis of AIRE expression in thymic epithelial cell (TEC; EpCAM$^+$CD45$^-$) subsets.

(b) 3-week-old C57BL/6 pups from control SPF and GF mothers and GF mothers treated with acetate in drinking water (ADW). **(a)** Immunofluorescence staining of thymus sections from age-matched untreated C57BL/6 mice showing AIRE expression in K14$^+$medulla and in β5t$^+$ cortical epithelial cells (cTEC; white arrows) situated at the cortico-medullary junction. β5t=cortical epithelial marker; K14=medullary epithelial cell (mTEC) marker; scale bars = 50μm.

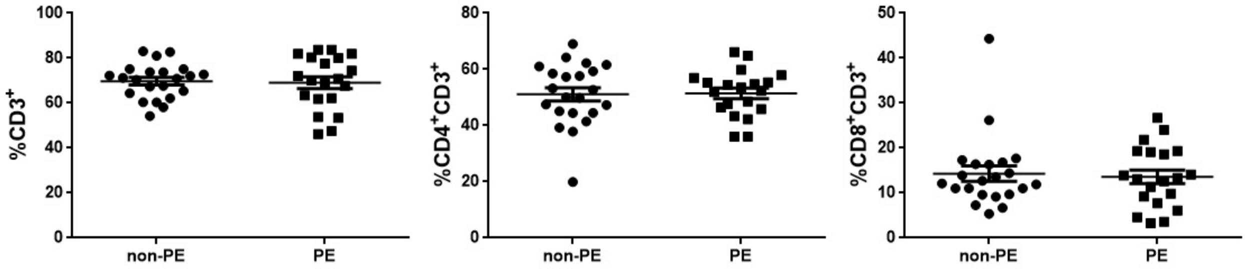

Supplementary Figure 6. The different subsets of fetal thymic T cells in normal and preeclamptic pregnancies

Scattered dot plots comparing the percentages (Mean ± SEM) of thymic T cells ($CD3^+$ cells), conventional CD4 and CD8 T cell subsets (from left to right) within lymphocytes in cord blood (n=22 for non-PE, n=20 for PE group).

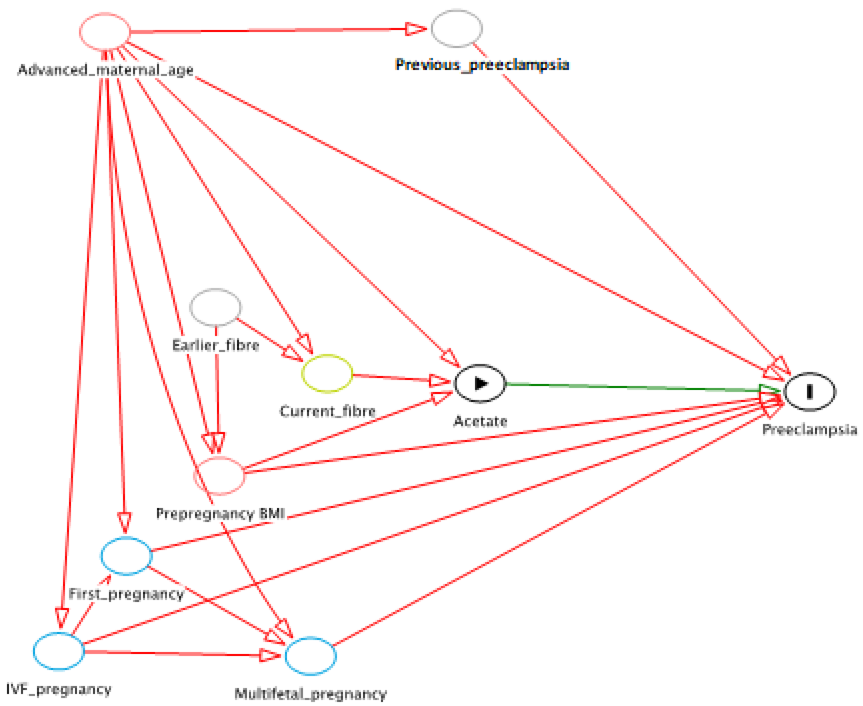

Supplementary Figure 7.

A directed acyclic graph (DAG) drawn from http://www.dagitty.net/ (DAGitty: A Graphical Tool for Analysing Causal Diagrams) to demonstrate the proposed causal relationship between the primary exposure (serum acetate), the primary outcome (preeclampsia) and relevant covariates. Nodes, or factors, are denoted by circles, and lines with an arrowhead are drawn to represent the direction of the relationship between the nodes (e.g. increased BMI is associated with increased risk of preeclampsia rather than vice versa). Red nodes = potential confounding factors; green nodes = ancestors of the exposure; grey nodes = unobserved factors; blue nodes = ancestors of the outcome. The DAG assists in selecting potential confounding variables to be included in the statistical model, on the basis of a temporally and biologically plausible proposed causal model. The potential confounding variables identified in this DAG were advanced maternal age and pre-pregnancy body mass index (BMI).

Supplementary Table 1a. Participant Characteristics of *Nepean cohort 1*

Characteristic	Control (n=50)	Preeclampsia (n=50)	P value
**Maternal**			
Maternal age (yr)	30 ± 6.1	29 ± 6.1	0.38
Caucasian ethnicity	45 (90%)	43 (86%)	0.54
Body Mass Index (kg/m^{-2})	24 (21-26)	28 (24-34)	**< 0.001**
Gestation at ultrasound (wk.)	32 (27-35)	34 (31-37)	**0.003**
Gestational diabetes	1 (2%)	4 (8%)	0.17
**Fetal**			
Ultrasound estimated fetal weight (g)	1894 ± 884	2412 ± 1097	**0.01**
Small for gestational age	5 (10%)	10 (20%)	0.19
Large for gestational age	4 (8%)	7 (3%)	0.20

Continuous variable presented as mean ± SD or median (Q1-Q3); categorical variables presented as numbers (percentage). Small for gestational age and large for gestational age were defined as an estimated fetal weight (EFW) less than 10th centile and more than 90th centile, respectively, based on Hadlock formula.

Supplementary Table 1b. T-test output of thymus measurements in control and preeclampsia group of *Nepean cohort 1*

Variable	Preeclampsia (mean)	Non-Preeclampsia (mean)	Mean difference (95% CI)	p-value
Volume (mL)	1.6±1.2	2.6±1.3	0.98 (0.49-1.46)	**0.0001**
MTD (cm)	2.9±0.8	3.3±0.8	0.49 (0.08-0.71)	**0.01**
APD (cm)	1.3	1.2	-0.008 (-0.15-0.13)	0.92
Circumference (mm)	75.5	82.1	6.64 (-1.151-14.80)	0.11
Area (cm^2)	348.6	402.4	54 (-9-117)	0.09

MTD: maximum transverse diameter, APD: anteroposterior diameter

Supplementary Table 2. Participant Characteristics *Nepean cohort 2*

Characteristic	Preeclampsia (24)	Non-Preeclampsia (863)	p-value
Maternal age (yr)	26.5 (22.0 - 33.0)	29.0 (25.0 - 32.0)	0.18
Nulliparity (%)	62.5	33.7	**0.007**
Body Mass Index (kg.m-2)	28.1 (24.4 - 34.9)	25.2 (22.1 - 30.0)	**0.03**
Fetal morphometry at ultrasound			
Gestation (wk.)	19.3 (19.0 - 19.9)	19.1 (18.9 - 19.6)	0.18
Thymus diameter (mm)	16.5 (2.1)	18.3 (2.2)	**0.0002***
Head circumference (mm)	166.6 (159.9 - 170.8)	162.5 (157.4 - 167.7)	**0.04**
Biparietal diameter (mm)	44.5 (42.5 - 45.8)	43.7 (42.0 - 45.4)	0.27
Abdominal circumference (mm)	142.9 (140.8 - 149.4)	141.6 (136.2 - 148.0)	0.11
Femur length (mm)	30.9 (29.5 - 32.0)	29.9 (28.5 - 31.3)	**0.02**
Gestation at delivery (wk.)	37.2 (34.4 - 38.8)	39.9 (38.9 - 40.7)	**<0.0001**

*Unpaired t-test expressed as Mean (SD). All other analyses used Mann-Whitney U-test, expressed as Median (Q1 – Q3).

Supplementary Table 3. Participant Characteristics of *Nepean cohort 3*

Characteristic	Preeclampsia (30)	Non-Preeclampsia (62)
Maternal age (yr)	28.9±6	30.3±4.8
Gestational week	35.4±3.2	39.2±0.9
Birthweight (g)	2527±1054	3558±451
Steroid treatment (%)	50	0

Supplementary Table 4. Participant Characteristics of *BIS cohort*

Characteristic	Preeclampsia (31)	Non-Preeclampsia (293)	p-value
Maternal Age (yr)	31.77±5.42	32.28±4.33	0.55
Previous pregnancies			0.005
No	14 (46.7%)	65 (23.0%)	
Yes	16 (53.3%)	217 (77.0%)	
BMI	27.81±5.70	25.60±5.45	0.060
Gestation week	37.58±2.25	39.16±1.42	<0.001
Birthweight (g)	3095.32±604.20	3562.69±536.41	<0.001
Steroid Treatment			0.57
No	31 (100.0%)	290 (99.0%)	
Yes	0 (0.0%)	3 (1.0%)	

Supplementary Table 5a. Participant Characteristics of *Autopsy cohort*

Characteristic	Preeclampsia (5)	Non-Preeclampsia (6)	p-value
Maternal age (yr)	32.2±6.26	31.33±3.78	0.773
Nulliparous	3 (60%)	4 (66.67%)	0.999
Gestational age (wk)	24.8±1.30	23.83±1.47	0.292
Birth weight (g)	394.6±70.35	655.2±231.9	0.058
Thymus weight (g)	0.28±0.13	1.33±0.70	**0.004**
Spleen weight (g)	0.52±0.22	1.3±0.80	**0.030**
Placental disc weight (g)	109.4±37.67	212±55.7	**0.032**

Supplementary Table 5b. T cell populations in the fetal thymus and spleen of *Autopsy cohort*

Variable	PE.1	PE.2	PE.3	PE.4	PE.5	p-value	n-PE.1	n-PE.2	n-PE.3	n-PE.4	n-PE.5	n-PE.6
Placental features of preeclampsia€	2	1	1	1	0	–	0	0	0	0	–	–
**Thymus Foxp3:**												
Expression*	0	0	0	0	0		1	0	1	1	1	0
Location^	0	0	0	0	0		2	0	2	2	2	0
Staining intensity#	0	0	0	0	0		2	0	2	2	2	0
**Thymus CD4:**												
Expression*	3	3	2	3	2		3	3	3	3	3	2
Location^	1	3	3	3	3		1	1	1	1	1	1
**Spleen Foxp3:**												
Expression*	0	0	0	0	0		1	0	1	1	1	0
Staining intensity#	0	0	0	0	0		1	0	1	2	2	0
**Spleen CD4:**												
Expression*	2	2	1	1	1		2	2	2	2	2	2
Staining intensity#	2	3	1	2	1		3	3	3	3	2	2

PE=Preeclampsia, n-PE=non-preeclamptic pregnancies; €(0=no; 1=yes with atherosis 2=decidual vessel vasculopathy without atherosis); *(0=0% of cells, 1=<10%, 2=10-75%, >3=>75%); ^(1=Cortex>Medulla, 2=Medulla>Cortex, 3=Medulla=Cortex, 4=Medulla only); #(0= absent; 1=weak, 2=moderate, 3=strong). αPlacental abruption, βIUGR, γSevere maternal sequelae of pre-eclampsia, δAscending infection (Group B streptococcus), εThanatophoric dysplasia Type II, ζDied <24 hours after delivery, ηSpondylocostal dysostosis, θUrorectal septum malformation, ιMicrocephaly.

Supplementary Table 6. Participant Characteristics of *Nepean cohort 4*

Characteristics	Preeclampsia (8)	Non-Preeclampsia (36)
Maternal age (yr)	28.3±8.0	31.3±4.6
Gestational age (yr)	35.5±3.4	38.6±1.6
Birthweight (g)	2549±1137	3353.3±574
