# Supplementary material for: Decreased maternal serum acetate and impaired fetal thymic and regulatory T cell development in preeclampsia
Source: Nat Commun. 2019 Jul 10;10:3031. doi: 10.1038/s41467-019-10703-1 (PMC6620275; doi:10.1038/s41467-019-10703-1)
Supplement: Supplementary file 3 — Reporting Summary [file 41467_2019_10703_MOESM3_ESM.pdf]

nature research

Corresponding author(s): Professor Ralph Nanan

Last updated by author(s): Apr 14, 2019

# Reporting Summary

Nature Research wishes to improve the reproducibility of the work that we publish. This form provides structure for consistency and transparency in reporting. For further information on Nature Research policies, see Authors & Referees and the Editorial Policy Checklist.

## Statistics

For all statistical analyses, confirm that the following items are present in the figure legend, table legend, main text, or Methods section.

| n/a | Confirmed | |
|---|---|---|
| ☐ | ☒ | The exact sample size (*n*) for each experimental group/condition, given as a discrete number and unit of measurement |
| ☐ | ☒ | A statement on whether measurements were taken from distinct samples or whether the same sample was measured repeatedly |
| ☐ | ☒ | The statistical test(s) used AND whether they are one- or two-sided<br>*Only common tests should be described solely by name; describe more complex techniques in the Methods section.* |
| ☐ | ☒ | A description of all covariates tested |
| ☐ | ☒ | A description of any assumptions or corrections, such as tests of normality and adjustment for multiple comparisons |
| ☐ | ☒ | A full description of the statistical parameters including central tendency (e.g. means) or other basic estimates (e.g. regression coefficient) AND variation (e.g. standard deviation) or associated estimates of uncertainty (e.g. confidence intervals) |
| ☒ | ☐ | For null hypothesis testing, the test statistic (e.g. *F*, *t*, *r*) with confidence intervals, effect sizes, degrees of freedom and *P* value noted<br>*Give P values as exact values whenever suitable.* |
| ☒ | ☐ | For Bayesian analysis, information on the choice of priors and Markov chain Monte Carlo settings |
| ☒ | ☐ | For hierarchical and complex designs, identification of the appropriate level for tests and full reporting of outcomes |
| ☐ | ☒ | Estimates of effect sizes (e.g. Cohen's *d*, Pearson's *r*), indicating how they were calculated |

*Our web collection on statistics for biologists contains articles on many of the points above.*

## Software and code

Policy information about availability of computer code

| | |
|---|---|
| Data collection | Thymus images were collected by US (Voluson 730 and E8; GE Healthcare, Zipf, Austria). Flowcytometry was performed by FACSVerse (8-channel, BD), FACSCalibur (3-channel, BD), FACSCanto II (8-channel, BD) or BD LSRII. Acetate levels were measured Bruker Avance III 600 MHz spectrometer equipped with a cryoprobe using automated data collection via IconNMR software (Bruker). |
| Data analysis | Thymus images were analysed by virtual organ computer-aided analysis (VOCAL) in Viewpoint software, version 5.6.12.601 (ViewPoint Bildverarbeitung GmbH, a GE Healthcare subsidiary). FlowJo (version 9 and 10) was used to analyze flowcytometry data. GraphPad Prism7 for Windows (GraphPad Prism, San Diego, CA), SAS v9.3 (SAS Institute Inc., Cary, NC, USA) or Stata 15.1 (StataCorp, College Station, TX, USA) were used for graphs and statistical analysis. |

For manuscripts utilizing custom algorithms or software that are central to the research but not yet described in published literature, software must be made available to editors/reviewers. We strongly encourage code deposition in a community repository (e.g. GitHub). See the Nature Research guidelines for submitting code & software for further information.

## Data

Policy information about availability of data

All manuscripts must include a data availability statement. This statement should provide the following information, where applicable:

- Accession codes, unique identifiers, or web links for publicly available datasets
- A list of figures that have associated raw data
- A description of any restrictions on data availability

The datasets generated during and/or analysed during the current study are available from the supporting excel spreadsheet. Other relevant information for human cohorts is available from corresponding author (Professor Ralph Nanan) upon reasonable request. There is no data with mandated deposition.

# Field-specific reporting

Please select the one below that is the best fit for your research. If you are not sure, read the appropriate sections before making your selection.

☒ Life sciences ☐ Behavioural & social sciences ☐ Ecological, evolutionary & environmental sciences

For a reference copy of the document with all sections, see nature.com/documents/nr-reporting-summary-flat.pdf

# Life sciences study design

All studies must disclose on these points even when the disclosure is negative.

| | |
|---|---|
| Sample size | To determine sample size, power analysis (power = 80 % and significance level = 5 %) was performed. Animal experiments were performed using 10 mice per group. |
| Data exclusions | No data were excluded from the analyses. |
| Replication | For thymus ultrasound findings, intra-observer reliability and Inter-rater reliability were assessed. All mouse experimental findings were successfully reproduced by each attempt. |
| Randomization | Human cohorts are not randomized due to the nature of the studies. Animals were randomly assigned to different groups. |
| Blinding | Investigators were not blinded. |

# Reporting for specific materials, systems and methods

We require information from authors about some types of materials, experimental systems and methods used in many studies. Here, indicate whether each material, system or method listed is relevant to your study. If you are not sure if a list item applies to your research, read the appropriate section before selecting a response.

## Materials & experimental systems

| n/a | Involved in the study | |
|---|---|---|
| ☐ | ☒ | Antibodies |
| ☒ | ☐ | Eukaryotic cell lines |
| ☒ | ☐ | Palaeontology |
| ☐ | ☒ | Animals and other organisms |
| ☐ | ☒ | Human research participants |
| ☒ | ☐ | Clinical data |

## Methods

| n/a | Involved in the study | |
|---|---|---|
| ☒ | ☐ | ChIP-seq |
| ☐ | ☒ | Flow cytometry |
| ☒ | ☐ | MRI-based neuroimaging |

## Antibodies

| | |
|---|---|
| Antibodies used | V500 anti-CD4 (BD, 562970, 1:40), APC-H7 anti-CD3 (BD, 560176, dilution 1:40), AF488 anti-Foxp3 (BioLegend, 320212, 1:50), V450-anti-Helios (Biolegend, 137220, 1.5:100), AF488-anti Foxp3 (BD, 560047, 1:20), PE-anti-CD4 (BD, 550630, 1:20), PE-Cy5.5-anti-CD45RA (BD, 555490, 1:20), Foxp3 (ab20034; 1:200, Abcam, Cambridge, UK), CD4 (NCL-L CD4-368, Novocastra 1/50 dilution), anti-mouse CD4 (Clone: RM4-5, Pacific Blue, BD Biosciences, 558107, 1:400 dilution), anti-mouse CD25 (Clone: PC61, PE Cy7, 552880, BD Biosciences, 1:400 dilution), anti-mouse Foxp3 (Clone: FJK-16s, FITC, eBioscience, 11-5773-82, 1:200 dilution), CD45 (30-F11, 1:400, BD Biosciences 557659), CD326/EpCAM (G8.8, 1:3000, eBioscience 25-5791-80), UEA-1 lectin (1:4000, Vector Labs B-1065), MHC Class II (1:5000, Biolegend 107620), Ly51 (1:2000, BD Biosciences 553735), AIRE (5G12, 1:100, eBioscience 3-5934-82), β5t (1:400, MBL PD021) and Keratin 14 (1:400, Biolegend 906004). |
| Validation | Validation statements for antibodies are available on the manufacturer's website. |

## Animals and other organisms

Policy information about studies involving animals; ARRIVE guidelines recommended for reporting animal research

| | |
|---|---|
| Laboratory animals | Germ-Free (GF) C57BL/6 mice were derived from Germ-Free Unit (Walter and Eliza Hall Institute of Medical Research). Pregnant GF mice from E18 were fed at libitum with sodium acetate (200mM, except where indicated) provided in the drinking water. Then 3-week-old pups (total, n=10) were studied. |
| Wild animals | The study did not involve wild animals. |
| Field-collected samples | The study did not involve the samples collected from the field. |

Ethics oversight | All experimental procedures involving mice were carried out according to protocols approved by the relevant Animal Ethics Committee of Monash University, Melbourne, Australia.

Note that full information on the approval of the study protocol must also be provided in the manuscript.

## Human research participants

Policy information about studies involving human research participants

Population characteristics | Population characteristics were provided in the supplementary table 1 to 6.

Recruitment | Participants who meet the inclusion criteria were explained and only recruited when written consents were given on volunteer basis.

Ethics oversight | All nepean cohorts were approved by The Ethics Committee of the Sydney West Area Health Service, Australia. The BIS cohort was approved by the Barwon Health Human Research and Ethics Committee. The fetal autopsy cohort was approved by the Health Human Research and Ethics Committee.

Note that full information on the approval of the study protocol must also be provided in the manuscript.

## Flow Cytometry

### Plots

Confirm that:

☒ The axis labels state the marker and fluorochrome used (e.g. CD4-FITC).

☒ The axis scales are clearly visible. Include numbers along axes only for bottom left plot of group (a 'group' is an analysis of identical markers).

☒ All plots are contour plots with outliers or pseudocolor plots.

☒ A numerical value for number of cells or percentage (with statistics) is provided.

### Methodology

Sample preparation | Mononuclear cells from the Nepean Cohorts (both peripheral blood and cord blood samples) were isolated using Ficoll-Hypaque (Amersham Pharmacia, Piscataway NJ) density gradient centrifugation. All samples were frozen at -196C for further flow cytometric analysis. BIS Cohort: Umbilical cord blood was collected by syringe and immediately diluted in 10IU/mL preservative-free sodium heparin (Pfizer) in 10ml of RPMI 1640 (Gibco, Life Technologies). Venous peripheral blood (PB) was collected at 6 months, 1 year and 4 years of age and added to a 15ml tube containing 10IU/mL preservative-free sodium heparin (Pfizer). All blood was processed within 18 hours of collection. Mononuclear cells (MNC) were isolated by density gradient centrifugation (Lymphoprep, Axis-Shield). Then cells were stained for analyses.

Instrument | Flowcytometry was performed by FACSVerse (8-channel, BD), FACSCalibur (3-channel, BD), FACSCanto II (8-channel, BD) or BD LSRII.

Software | FlowJo (version 9 and 10) was used to analyze flowcytometry data.

Cell population abundance | Cell sorting was not conducted for this study.

Gating strategy | All gating strategies have been provided either in the figure or in the supplementary information.

☒ Tick this box to confirm that a figure exemplifying the gating strategy is provided in the Supplementary Information.
